# Amelioration of acute lung injury by *Salvia miltiorrhiza*-derived extracellular vesicles: through repair of the vascular barrier and modulation of lung microbiota

**DOI:** 10.1186/s13020-025-01203-0

**Published:** 2026-01-06

**Authors:** Jiawang Huang, Zhiying Feng, Jingmin Fu, Junju Zou, Qin Xiang, Xiu Liu, Ling Li, Rong Yu

**Affiliations:** 1 School of Traditional Chinese Medicine, Hunan University of Chinese Medicine, Changsha, Hunan 410208 People’s Republic of China; 2 Academy of Chinese Medical Sciences, Hunan University of Traditional Chinese Medicine, Changsha, Hunan 410208 People’s Republic of China

**Keywords:** *Salvia miltiorrhiza*-derived extracellular vesicles, Acute lung injury, pulmonary vascular barrier, lung microbiota

## Abstract

**Background:**

Acute lung injury (ALI) is a severe respiratory disease characterized by diffuse lung injury, vascular barrier dysfunction, and inflammatory responses. Its current treatments such as corticosteroids often involve adverse effects, highlighting the need for alternative therapies. *Salvia miltiorrhiza*-derived extracellular vesicles (SMEVs) have shown a potential therapeutic value for ALI due to their anti-inflammatory and barrier-protective properties, but the specific mechanisms remain unclear.

**Methods:**

SMEVs were extracted and purified through differential centrifugation coupled with sucrose density gradient centrifugation, and were analyzed by transmission electron microscopy (TEM) and nanoparticle tracking analysis (NTA). Biosafety assessment was then conducted in zebrafish embryos, mouse organs, and human umbilical vein endothelial cells (HUVEC). Subsequently, the treatment efficacy of SMEV on LPS-induced HUVEC inflammation was evaluated in vitro. LPS-induced ALI mice were then treated with SMEVs to further evaluate the posttreatment lung histopathology, vascular barrier markers, and microbial composition using metagenomics in vivo.

**Results:**

SMEVs exhibited a typical bilayer structure (average size: 177.7 nm) and excellent biosafety properties. In vitro, SMEVs effectively reduced LPS-induced inflammation (IL-1β, IL-6, TNF-α) and promoted wound healing in HUVEC, while in vivo, SMEVs ameliorated pulmonary edema and inflammation, and restored the VE-cadherin expression. Metagenomic analysis revealed that SMEVs were capable of regulating lung microbiota and reducing the pathogenic bacterial (e.g., g-Listeria, g-Streptococcus) and microbial diversity and richness after LPS stimulation.

**Conclusion:**

SMEVs can ameliorate ALI by repairing the vascular barrier and modulating lung microbiota, offering a novel therapeutic strategy for this disease. Future research may focus on the SMEV-microbiota-immune interaction targeting ALI treatment.

## Introduction

Acute lung injury (ALI) is a clinically common and severe respiratory disease with high morbidity and mortality worldwide [1]. The key pathological features of ALI include diffuse lung injury, vascular barrier dysfunction, and inflammatory responses; in these conditions, pulmonary microvascular endothelial cells, which constitute approximately half of the lung cell population, play a crucial role [2]. Disruption of the vascular barrier system can lead to increased vascular permeability, forcing large amounts of exudates, inflammatory cells, and inflammatory factors to infiltrate into lung interstitial tissues, which may induce pulmonary edema and exacerbate lung dysfunction [3, 4]. The pulmonary vascular endothelial barrier is primarily composed of adherens junctions and tight junctions. Formation of the former in endothelial cells fundamentally depends on the vascular endothelial-cadherin (VE-cadherin) [5]. Once VE-cadherin is disrupted, cytoskeletal rearrangement occurs, leading to impaired adherens junctions that may further induce endothelial barrier dysfunction and increase endothelial permeability [6]. The lung microbiota, an essential component of the human microbiota, consists of the entire microbial community in the lungs, including bacteria, viruses, and fungi. It forms a biological system that interacts with the host’s lung microenvironment through cellular signaling pathways and metabolites, creating a mutual influence [7]. The establishment of lung microbiota is a critical factor in developing a mature lung immune system and protecting the lungs from harmful inflammatory responses. This not only contributes to normal respiratory tract development, but also plays multiple roles in maintaining human health, including regulating respiratory immunity and maintaining respiratory health by preventing pathogen spread [8]. Clinical studies have shown that lung microbiota is related to the onset and progression of a range of lung diseases, and the crosstalk between the host’s pathophysiological changes and lung microbiota is pivotal in regulating the corresponding disease conditions [9]. In previous research, significant changes in microbial composition were observed in the lungs of LPS-induced rodent ALI models. Antibiotic treatment has been demonstrated to exert therapeutic effects against ALI by altering the composition and richness of lung microbiota [10], though the underlying mechanisms remain incompletely understood.

The current therapeutic strategies for ALI mainly include mechanical ventilation, pharmacological treatments (such as anti-inflammatory therapy), fluid management, and supportive care, all with significant limitations. Specifically, mechanical ventilation, while critical for maintaining oxygenation, may induce ventilator-associated lung injury (VILI) in case of excessive ventilation pressure and tidal volume, exacerbating the mechanical damage to alveoli and capillaries and even amplifying inflammatory cascade responses [11]. As for pharmacological treatments, corticosteroids are commonly used in clinical practice for suppressing excessive inflammatory responses, but their nonspecific immunosuppression may increase the risk of secondary infections and impose a negative impact on tissue repair with prolonged use [12]. Fluid management, though essential, presents a clinical dilemma: excessive volume loading worsens pulmonary edema, while restrictive strategies may compromise organ perfusion. This issue necessitates dynamic adjustments under meticulous monitoring procedures to address, posing a tough clinical challenge [13]. Moreover, most of the current therapeutic approaches merely focus on symptom control and life support, lacking targeted interventions for alveolar-capillary barrier repair or precise inflammatory regulation, resulting in suboptimal efficacy and high mortality. In view of the above clinical context, developing safer, more targeted ALI therapies has become an urgent research priority.

Traditional Chinese medicine (TCM) represents a treasure of Chinese culture. *Salvia miltiorrhiza* (“Danshen” in Chinese), as a traditional Chinese herbal medicine, has been widely used in TCM clinical practice owing to its antioxidant, neuroprotective, anti-fibrotic, anti-inflammatory, and anti-tumor properties [14]. According to modern pharmacological studies, the main active compounds in the roots of *Salvia miltiorrhiza* comprise lipophilic diterpenoids (primarily tanshinones) and hydrophilic phenolic compounds (primarily salvianolic acids). Specifically, tanshinones are a group of abietane-type diterpenoids, including dihydrotanshinone I, tanshinone I, cryptotanshinone, and tanshinone IIA. These compounds share related biological activities and present with common antioxidant, anti-inflammatory, antibacterial, and anti-tumor properties [15, 16]. Salvianolic acids, particularly salvianolic acid A and salvianolic acid B, exhibit potent antioxidant capabilities attributed to their polyphenolic structures, along with effects of anti-inflammation, immune regulation and reduction of endothelial cell adhesion [17]. Furthermore, *Salvia miltiorrhiza* was also reported to mitigate inflammatory responses by interfering with the activation of NF-*κ*B signaling pathway, thereby effectively inhibiting SARS-CoV-2-induced ALI in mouse models [18].

In recent years, nanoscale extracellular vesicles (EVs) that are actively released by plant cells have attracted widespread attention in clinical research. More specifically, the nanoscale EVs derived from Chinese herbal medicines are revealed to play a vital role in intercellular and interspecies communication by facilitating information and substance transfer [19]. By encapsulating the bioactive compounds derived from Chinese herbs within their homologous plant-derived EVs, the bioavailability of some poorly soluble active ingredients can be substantially enhanced. Moreover, the structure of these EVs can be further modified to enable targeted delivery of effective drug components or to exert synergistic effects among multiple components [20, 21]. Earlier studies have shown that the EVs derived from plants like ginseng, ginger, kudzu, and dandelion are capable of alleviating inflammatory diseases, inhibiting tumor proliferation, and preventing hypertension [22, 23]. In this work, on the basis of existing findings, we prepared and characterized *Salvia miltiorrhiza*-derived EVs (SMEVs), evaluated their biosafety, and investigated their therapeutic effects on LPS-induced ALI through comprehensive in vitro and in vivo experiments. Our objective was to provide in-depth evidence for the use of *Salvia miltiorrhiza* in ALI treatment.

## Materials and methods

### Experimental reagents

The reagents used in this study include: DMEM basal medium (Procell, WHB824D281), phosphate buffer solution (Procell, WHB824A031), and penicillin–streptomycin mixture (Procell, WH1021A161); fetal bovine serum (Thermo Fisher, SA210518); cell-specific lipopolysaccharide (Sigma-Aldrich, LPS-L2630, 0000155608) and animal-specific lipopolysaccharide (Sigma-Aldrich, LPS-L2800, 0000189843); CCK8 cell proliferation and cytotoxicity assay kit (Biosharp, BS350A); BCA protein quantification kit (Lianke Biotech, 81910335A); mRNA reverse transcription primers and PCR (real-time quantitative polymerase chain reaction, RT-qPCR) primers (synthesized by Shanghai Sangon Biotech Co., Ltd.); CD81 antibody (Servicebio, GB113151), VE-cadherin antibody (Servicebio, GB14013), fluorescein isothiocyanate isomer (FITC)-labeled donkey anti-mouse secondary antibody (Servicebio, GB22401), and CY3-labeled goat anti-rabbit secondary antibody (Servicebio, GB21303); 4′,6-diamidino-2-phenylindole (DAPI) solution and tetramethylrhodamine isothiocyanate (rhodamine phalloidin) (Beijing Solarbio Science & Technology Co., Ltd.).

### Experimental animals and cells

Male C57BL/6 J mice, aged 6–8 weeks with a body weight of 18–22 g, were purchased from Hunan Slack Jingda Laboratory Animal Co., Ltd. (animal quality license number: No. 430727231103210572), and were housed at the Animal Experiment Center of Hunan University of Chinese Medicine. The wild-type AB strain of zebrafish was obtained from the National Zebrafish Resource Center of China and maintained in a zebrafish breeding system at the Key Laboratory of Hunan Province at Hunan University of Chinese Medicine (developed by Beijing Aisheng Technology Development Co., Ltd.). Zebrafish embryos at 1 day post-fertilization, which were derived from natural spawning of adult wild-type AB strain zebrafish, were used in the experiment. All animal experiments were approved by the Animal Ethics Committee of Hunan University of Chinese Medicine (approval number: HNUCM21-2310-12). The human umbilical vein endothelial cell line (HUVEC) was sourced from the Cell Bank of the Chinese Academy of Sciences.

### Isolation, purification, and characterization of SMEVs

#### Isolation and purification of SMEVs

Fresh *Salvia miltiorrhiza* juice was subjected to differential centrifugation at 2000 × *g* for 20 min first and then at 10,000 × *g* for 60 min at 4 °C (Thermo, United States). The supernatant was collected and filtered through a 0.45 μm membrane, followed by ultracentrifugation at 150,000 × *g* for 90 min at 4 °C (BECKMAN COULTER, United States). The resulting pellet was resuspended in PBS and further purified through sucrose density gradient centrifugation (8%, 30%, 45%, and 60%) at 150,000 × *g* for 90 min at 4 °C. Lastly, the bands between 8%–30% and 30%–45% sucrose layers were collected, washed with PBS, and resuspended to obtain purified SMEVs.

#### Observation of SMEV morphology by transmission electron microscope (TEM)

SMEV suspension was placed onto a copper grid, allowing it to stand for 3–5 min. A 3% phosphotungstic acid solution was then added for staining for 5 min. After air-drying, the samples were observed (images captured) under a TEM (Hitachi, Japan).

#### Nanoparticle tracking analysis (NTA)

The obtained SMEV precipitate was resuspended and mixed in PBS for NTA particle size detection at room temperature (NanoSight, United Kingdom).

#### BCA assay for SMEV protein concentration

The standard solution and BCA working solution were prepared, respectively, according to the manufacturer’s instructions. Then, 200 μL of BCA working solution and 20 μL of either the standard solution or SMEV sample were added to each well of a 96-well plate. The plate was incubated in the dark for 30 min at 37 °C, and the protein concentration was measured using a microplate reader at the wavelength of 562 nm (BioTek, United States).

#### Coomassie brilliant blue staining

SMEV proteins were extracted using RIPA lysis buffer. After denaturation, 4 × SDS polyacrylamide gel electrophoresis was performed. Then, 10% SDS-PAGE gel was cut, stained with Coomassie blue for 2 h, and rinsed 2–3 times, each for 1–2 h, using a rinse solution containing 2250 mL of 95% ethanol, 250 mL of glacial acetic acid, and 2500 mL of distilled water. Lastly, the gel was analyzed using a gel imaging system.

#### Agarose gel electrophoresis detection

Agarose gel was poured into the electrophoresis tank for solidification for 30 min at room temperature. Concurrently, a 5 μL RNA sample was mixed with 1 μL of loading buffer and 1 μL of dye, and the mixture was then loaded into each well of the plate. Subsequently, 1 × TAE buffer was added to the tank until submerging the gel, and electrophoresis was performed at 120 V for 60 min. Thereafter, the bands were observed and recorded using a UV gel imaging system.

#### miRNA sequencing analysis

Total RNA was extracted from SMEVs to prepare the miRNA library. The constructed library was sequenced on the Illumina Hiseq 2000/2500 platform, with a single-end read length of 50 bp (SE50). Then, miRNA data analysis was performed using the ACGT101-miR (v4.2) software.

#### Analysis of chemical compounds in SMEV aqueous extract by LC–MS/MS

LC–MS/MS analyses were performed using a UHPLC system equipped with a Phenomenex Kinetex C18 column (2.1 mm × 100 mm, 2.6 μm) coupled to an Orbitrap Exploris 120 mass spectrometer. The mobile phase consisted of (A) water containing 0.01% acetic acid and (B) a 1:1 (v/v) mixture of isopropanol (IPA) and acetonitrile (ACN). The auto-sampler temperature was maintained at 4 °C with an injection volume of 2 μL. The Orbitrap Exploris 120 mass spectrometer was operated in data-dependent acquisition (DDA) mode controlled by Xcalibur software. Electrospray ionization (ESI) source parameters were optimized as follows: sheath gas flow rate at 50 arbitrary units (Arb), auxiliary gas flow rate at 15 Arb, capillary temperature at 320 °C, full MS resolution at 60,000, and MS/MS resolution at 15,000. The collision energy was set using stepped normalized collision energy (SNCE) at 20/30/40, with spray voltages of 3.8 kV (positive ion mode) and − 3.4 kV (negative ion mode).Compound identification was based on three criteria: (1) components listed in the 2020 edition of Chinese Pharmacopoeia, (2) bioactive compounds reported in literature as being abundant in the studied materials, and (3) compounds previously documented in human blood samples according to literature reports [24–28].

### Biosafety and stability assessment of SMEVs

#### Zebrafish experiment

Zebrafish embryos were randomly selected and exposed to SMEVs at gradient concentrations from 50 to 500 μg/mL [21], with an untreated group set as control. The embryos were cultured at 28 ± 2 °C and observed until 96 h post-fertilization, during which the mortality and malformation rates of larvae were recorded.

#### CCK8 assay for cell viability

HUVEC cells were seeded in 96-well plates (4 × 10^3^ cells/well) and cultured for 24 h. The cells were then treated with SMEVs at seven gradient concentrations (0, 10, 50, 100, 150, 200, 500 μg/mL) and incubated for an additional 24 h. Following instructions of the CCK8 kit, the absorbance was measured at the wavelength of 450 nm (BioTek, United States), and the relative viability rate of cells (%) was calculated by: (mean absorbance of the experimental group ÷ mean absorbance of the control group) × 100%.

#### H&E staining for observing the toxic effects of SMEVs on mouse organs

Six C57BL/6 J mice were divided into a control group (Con) and an SMEV group (SMEV). The mice in each group were orally administered with equal volume of PBS or SMEVs (25 mg/kg) [29], respectively, for three consecutive days. Subsequently, the heart, lung, liver, spleen, and kidney tissues were collected from each mouse and fixed in 4% paraformaldehyde. The tissues were then dehydrated by the ethanol gradient method, cleared in xylene, embedded in paraffin, and sectioned for H&E staining. Lastly, the sections were dehydrated, cleared, mounted, and observed under a microscope.

#### Zeta potential analysis

The obtained SMEV precipitate was resuspended and mixed in ultrapure water for zeta potential detection at room temperature (Zetasizer Nano-ZS90, United Kingdom).

### Cell experiments

#### Cell culture and grouping

HUVEC cells were seeded in 6-well plates at a density of 1 × 10^6^ cells per well and divided into the Con group, LPS group, and SMEV group. After 24 h of culture, the LPS group was treated with 1 μg/mL LPS, while the SMEV group was treated with both 1 μg/mL and SMEVs to establish cell models. The cells were collected after 24 h of treatment.

#### CCK-8 assay

HUVEC cells were seeded in 96-well plates at a density of 4 × 10^3^ cells per well and divided into the Con group, LPS group, and SMEV group (at seven gradient concentrations: 20, 40, 60, 80, 100, 150, and 200 μg/mL). The LPS group was treated with 1 μg/mL LPS, while the SMEV group was treated with both 1 μg/mL and SMEVs to establish cell models. After 24 h of treatment, CCK-8 assay was performed following the same steps as described in Sect. “CCK8 assay for cell viability”.

#### SMEV uptake detection

SMEVs were labeled with the fluorescent dye PKH67 following the manufacturer’s instructions. The labeled SMEVs were co-incubated with HUVEC cells after adhesion for 24 h. The cells were then fixed, stained with Phalloidine rhodamine for cytoskeleton visualization, and counterstained with DAPI for nucleus. Imaging was performed using a confocal laser microscope (Nikon, Japan).

#### Scratch assay

HUVEC cells were seeded in 6-well plates at a density of 1 × 10^6^ cells per well and divided into the Con group, LPS group, and SMEV group. The LPS and SMEV groups were treated as described above. After 24 h of treatment, a straight scratch was made in the monolayer of the cells using a sterile pipette tip. The cells were washed three times with PBS to remove detached cells, and were then added with low-serum medium. The scratch healing process was photographed under a microscope at 0, 6, 12, and 24 h, and the wound healing rate was calculated by measuring the scratch width using Image J software.

#### RT-qPCR for mRNA expression

Total RNA was extracted from each group of cells by employing the Trizol method. According to manufacturers’ instructions, 1 μg of RNA was reverse-transcribed into cDNA using a reverse transcription kit, followed by amplification using a fluorescent quantitative PCR kit. The specific reaction conditions were as follows: pre-denaturation for 30 s at 95 °C, denaturation for 10 s at 95 °C, and annealing and extension for 30 s at 60 °C, for a total of 40 cycles (Bio-Rad, United States). Taking GAPDH as internal reference, the relative expression of target genes was calculated using the 2^−ΔΔCt^ method. The primer sequences are listed in Table 1.

**Table 1:** Primer sequences for cell experiments

Name	Primer sequence (5′-3′)
h-TNF-*α*-F	CTCATCTACTCCCAGGTCCTCTTC
h-TNF-*α*-R	CGATGCGGCTGATGGTGTG
h-IL-6-F	GTGTTGCCTGCTGCCTTCC
h-IL-6-R	TCTGAAGAGGTGAGTGGCTGTC
h-IL-1*β*-F	GCACCTGTACGATCACTGAACTG
h-IL-1*β*-R	CACTTGTTGCTCCATATCCTGTCC
h-GAPDH-F	CGACCAAATCCGTTGACTCC
h-GAPDH-R	CCTGTTCGACAGTCAGCCG

### Animal experiments

#### Preparation and intervention of ALI model mice

After acclimatization for one week, ALI model mice were established using C57BL/6 J mice through intratracheal instillation of 5 mg/kg LPS. Then, the mice were randomly divided into a model group (LPS) and an SMEV intervention group (SMEV); concurrently, a control group (Con) was set up through intratracheal instillation with an equal volume of pure water. Six h after modeling, the SMEV group was administered with SMEVs at the dosage of 25 mg/kg through gavage for 3 consecutive days, while the control group was treated with an equal volume of PBS. The body weight and general conditions of the mice were recorded throughout.

#### Observation of histopathological changes in lung tissues by H&E staining

Lung tissues were taken from each group of mice for H&E staining and observation, following the same procedure as described in Sect. “H&E staining for observing the toxic effects of SMEVs on mouse organs”.

#### Detection of mRNA expression in lung tissues by RT-qPCR

Total RNA was extracted from the lung tissues of each group of mice by employing the Trizol method. Subsequent steps followed Sect. “RT-qPCR for mRNA expression”, with primer sequences listed in Table 2.

**Table 2:** Primer sequences for animal experiments

Name	Primer sequence (5′-3′)
m-TNF-*α*-F	GGTGTTGCCTGCTGCCTTCC
m-TNF-*α*-R	GTTCTGAAGAGGTGAGTGGCTGTC
m-IL-6-F	CTGGCGGAGGAGGTGCTCTC
m-IL-6-R	GGAGGAAGGAGAAGAGGCTGAGG
m-IL-1*β*-F	GGTGTTGCCTGCTGCCTTCC
m-IL-1*β*-R	GTTCTGAAGAGGTGAGTGGCTGTC
m-GAPDH-F	GCCTCCTCCAATTCAACCCTTA
m-GAPDH-R	TTGTCTACGGGACGAGGAAAC

#### Detection of the expression of vascular endothelial marker protein CD31 and vascular endothelial barrier marker protein VE-cadherin in lung tissues by immunofluorescence assay.

Lung tissue sections were deparaffinized, rehydrated, and subjected to antigen retrieval using microwave. The sections were then blocked with serum for 30 min. Subsequently, both the primary antibodies and fluorescent FITC/CY3 secondary antibodies were incubated in the dark at room temperature for 1 h first and further at 4 °C overnight. The DAPI working solution was applied for nuclear counterstaining and incubated in the dark at room temperature for 10 min. Lastly, the sections were mounted and imaged under a fluorescence microscope.

### Metagenomic analysis of microbial community changes in lung tissues

Total DNA was extracted from the lung tissues of each group of mice and quantitatively analyzed. The DNA integrity was assessed by agarose gel electrophoresis. A paired-end library was then constructed through the following steps in sequence: fragmentation of genomic DNA by sonication, end repair of DNA fragments, addition of an “A” base to the 3′ end of DNA fragments, ligation of sequencing adapters, fragment selection, and PCR amplification. After quality control, high-throughput sequencing was performed on the NovaSeq 6000 platform in a PE150 sequencing mode. The raw sequencing data were preprocessed to remove adapters and low-quality sequences first, and de novo assembly was then conducted for each sample. The assembled contigs were subjected to CDS prediction, with short contig sequences being filtered out. Subsequently, a non-redundant Unigenes set was obtained through sequence clustering, and the Unigenes were aligned against the NR_meta database to acquire the taxonomic classification information at different levels. Further, Alpha and Beta diversity analyses were performed based on species-level annotations. Lastly, the Unigenes were compared against multiple functional databases using the DIAMOND software, and the differences in microbial composition among different groups were evaluated through LEfSe analysis and MetagenomeSeq differential analysis.

### Statistical analysis

All data were expressed as mean ± standard deviation (x̄ ± s). The homogeneity of variance was tested first before statistical analysis. For homogeneous variance, One-Way ANOVA with the Least-Significant Difference (LSD) method was used for comparisons between groups. For non-homogeneous variance, non-parametric rank-sum tests were applied (Kruskal–Wallis H test for the comparison of overall differences, followed by Mann–Whitney U test for pairwise comparisons). A P-value of < 0.05 or < 0.01 was considered statistically significant. Statistical graphs were plotted using GraphPad Prism. Bioinformatic analysis was performed using OmicStudio tools at https://www.omicstudio.cn/tool. Heatmaps (or other graphics) were generated using R (https://www.r-project.org/) on the OmicStudio platform (https://www.omicstudio.cn/tool).

## Results

### Isolation, purification, and characterization of SMEVs

**Fig. 1:**
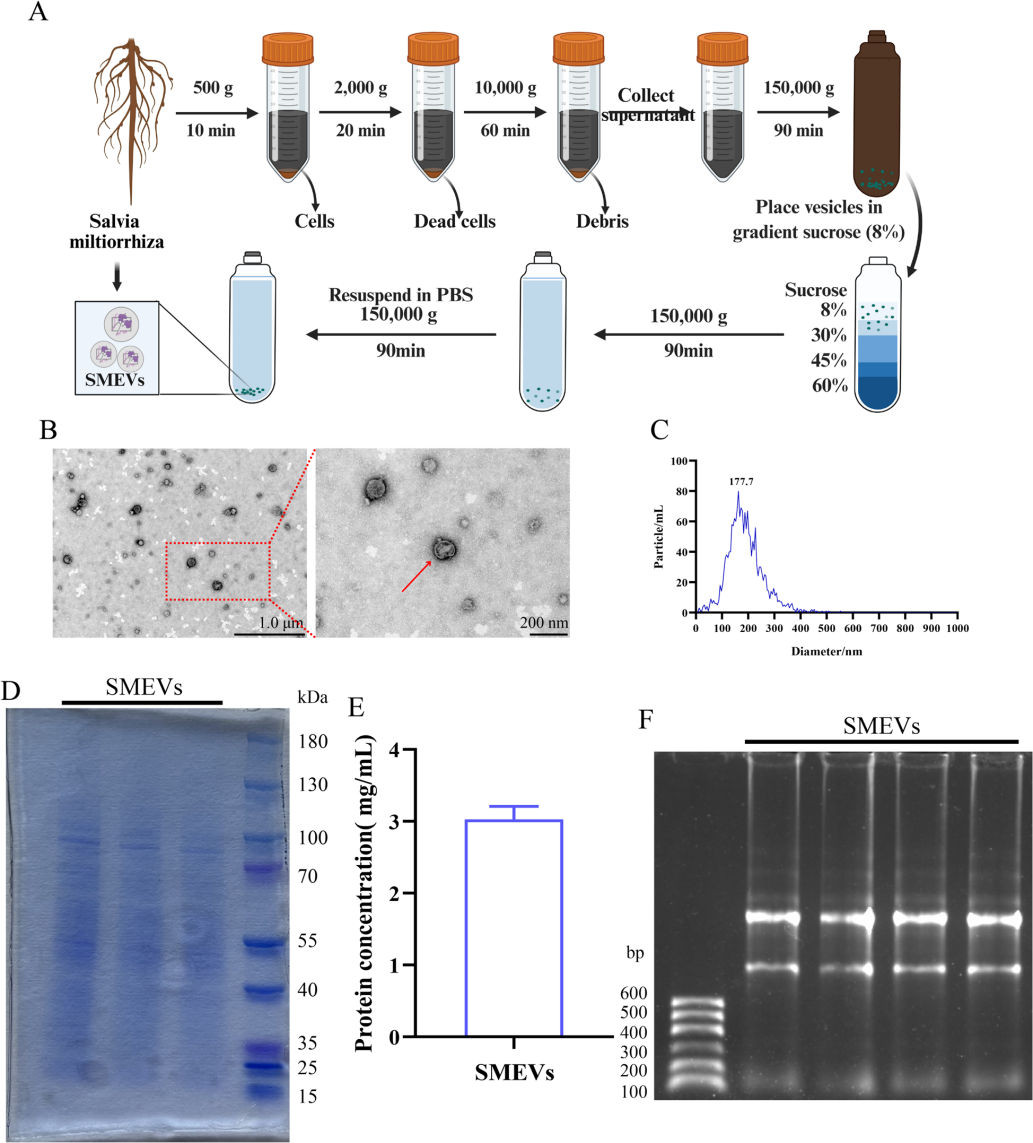
Isolation, Purification and Structural Characterization of SMEVs. **A** Schematic diagram of the extraction and purification protocol for SMEVs. **B** A representative TEM image of SMEVs. **C** Size distribution profile of SMEVs determined by dynamic light scattering (DLS). **D** SDS-PAGE analysis of the protein composition in SMEVs. **E** Protein concentration quantification of SMEVs by bicinchoninic acid (BCA) assay. **F** Agarose gel electrophoresis of the nucleic acid content in SMEVs

**Fig. 2:**
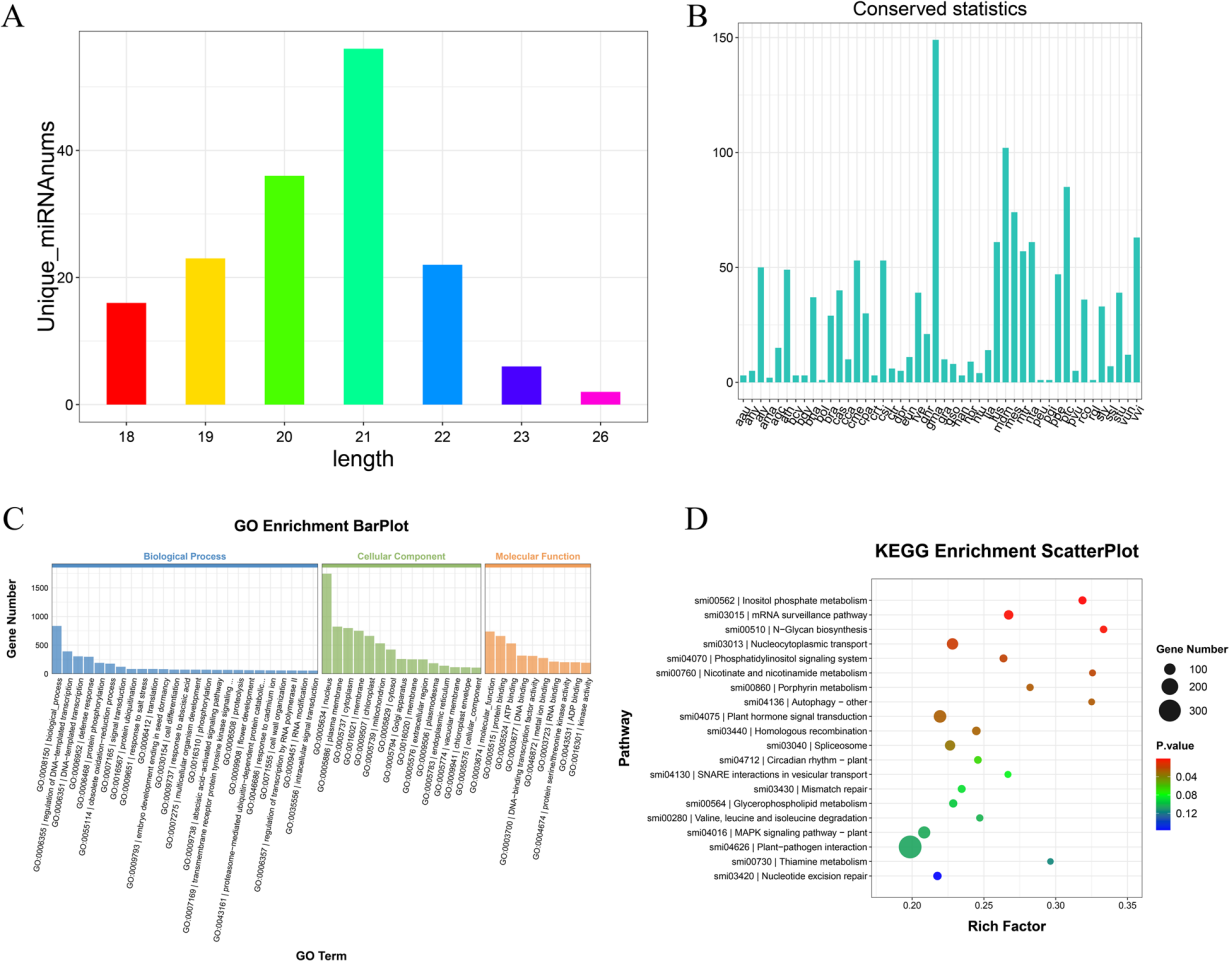
Characterization of SMEVs by miRNA Sequencing. **A** The miRNA count in SMEVs. **B** Conservation analysis of miRNA. **C** GO functional analysis results based on SMEV miRNA sequencing. **D** KEGG analysis results based on SMEV miRNA sequencing

SMEVs were extracted and purified through differential centrifugation combined with sucrose density gradient centrifugation (Fig. 1A). The TEM findings revealed that the extracted SMEVs exhibited a typical bilayer membrane structure with a spherical or oval morphology (Fig. 1B). The NTA results demonstrated a homogeneous size distribution, with an average size of 177.7 nm (Fig. 1C). The protein concentration was detected to be 3.0 mg/mL by BCA assay, suggesting high abundance of proteins and nucleic acids in *Salvia miltiorrhiza*-derived SMEVs (Fig. 1E). Protein electrophoresis and nucleic acid agarose gel electrophoresis confirmed the presence of proteins and RNA components in the SMEVs (Fig. 1D, Fig. 1F). Sequencing analysis of miRNAs identified 161 miRNAs in SMEVs, primarily ranging from 18 to 26 bp in length (Fig. 2A). Subsequently, a conservation analysis of miRNAs across selected species was conducted (Fig. 2B). Functional enrichment analysis showed that the miRNAs carried by SMEVs were predominantly involved in signal transduction and metabolic regulation, indicating that SMEVs may exert pharmacological effects through vesicle-mediated delivery mechanisms.

### Safety and stability evaluation of SMEVs

**Fig. 3:**
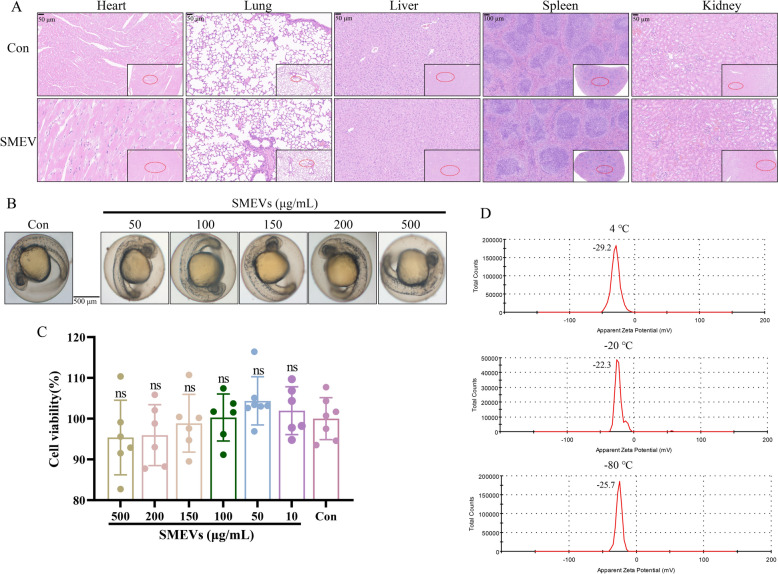
Safety Assessment of SMEVs. **A** H&E staining images of heart, lung, liver, spleen, and kidney tissue sections from normal control and SMEV-treated mice. **B** Morphological assessment of zebrafish embryos treated with SMEVs at varying concentrations. Scale bar: 500 μm. **C** Cell viability evaluation by CCK-8 assay (n = 6, x̅ ± s). **D** Zeta potential measurements of SMEVs stored under varying temperature conditions

Mice were administered with SMEVs at a dosage of 25 mg/kg through oral gavage for three consecutive days. Histopathological examination showed intact tissue structures with no significant pathological changes in major organs, including heart, lungs, liver, spleen, and kidneys (Fig. 3A). In zebrafish embryo development models, it was found that exposure to SMEVs (at all concerned concentrations) did not increase the embryo mortality or developmental malformations (Fig. 3B). CCK-8 assays demonstrated that SMEVs had no significant impact on the viability of HUVEC cells (Fig. 3C). To further assess the stability of SMEVs, zeta potential measurements were performed on SMEVs at 4 °C, − 20 °C, and − 80 °C, respectively, for 10 days (Fig. 3D). Collectively, these comprehensive analyses demonstrate that SMEVs exhibit favorable biocompatibility, minimal systemic toxicity, and relatively high stability.

### Analysis of chemical constituents in SMEVs by LC–MS/MS

**Fig. 4:**
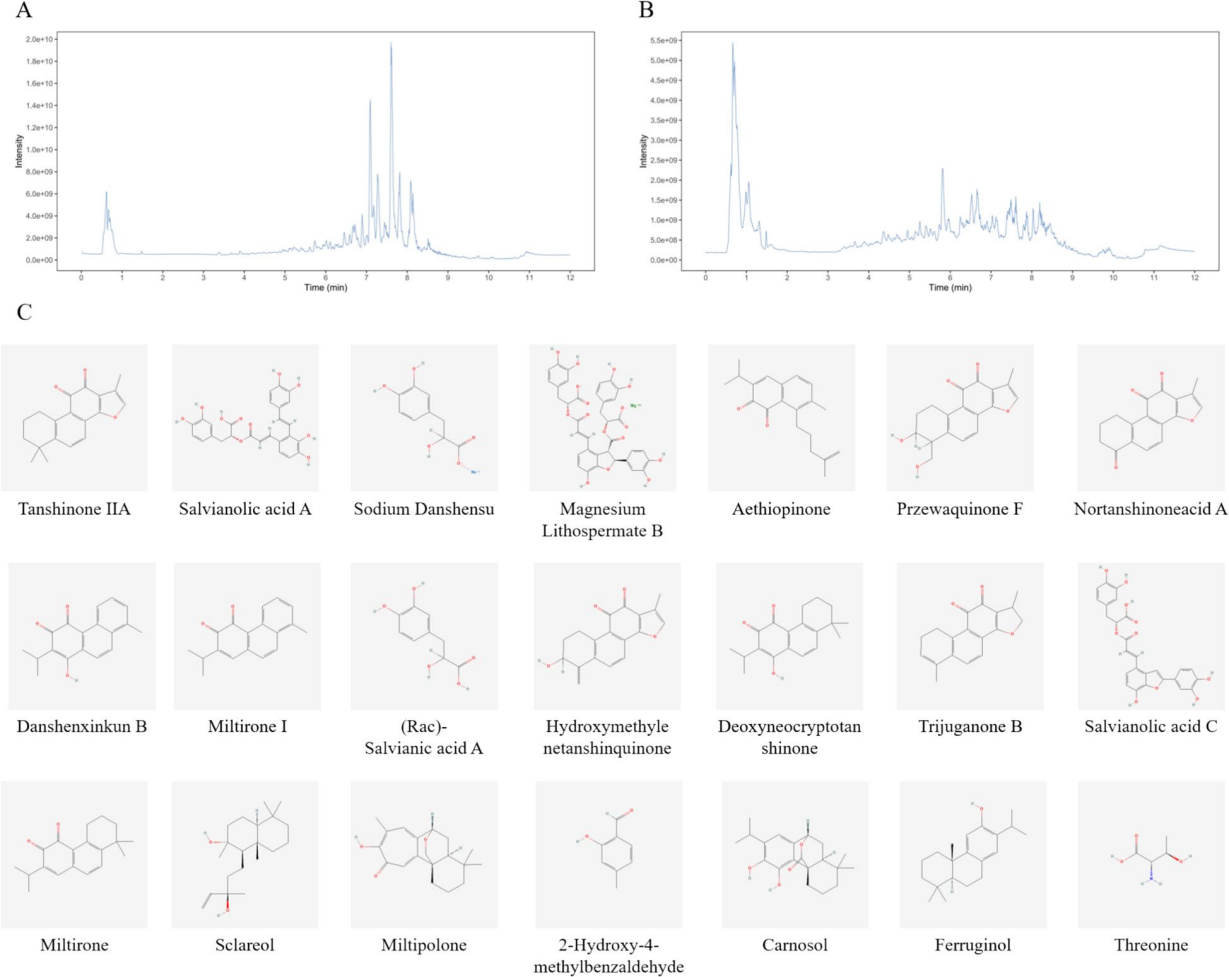
Detection of components in SMEVs by LC–MS/MS. **A** Base peak chromatogram of SMEVs in the positive ion mode. **B** Base peak chromatogram of SMEVs in the negative ion mode. **C** The main monomers of *Salvia miltiorrhiza* in SMEVs

A total of 1560 compounds (including 328 terpenoids, 137 alkaloids, 92 flavonoids, 69 Oxo fatty acids, 86 lignans, 62 coumarins, 46 phenolic acids, 43 aldehydes, 29carbohydrate, 27 quinones, 17 amino acids, 9 steroids, and 615 other compounds) were identified through comparison with reference standards, TCM databases, and literature data, with 738 and 822 compounds being detected in positive and negative ion modes, respectively. The total ion current chromatograms are presented in Fig. 4A, B. Combined with the results of LC–MS/MS and other related references (including 2020 edition of Chinese Pharmacopoeia, bioactive components documented or reported to be abundant in literature, and components in human blood reported in literature), a total of 21 *Salvia miltiorrhiza* related active substances and chemical substances were identified from SMEVs, including Tanshinone IIA, Aethiopinone, Przewaquinone F, 2-Isopropyl-8-methylphenanthrene-3,4-dione, Miltipolone, Danshenxinkun B, Deoxyneocryptotanshinone, Carnosol, Ferruginol, Salvianolic acid A, Sodium Danshensu, Magnesium Lithospermate B, Nortanshinone, Hydroxymethylenetanshinquinone, Trijuganone B, Salvianolic acid C, (Rac)-Salvianic acid A, 2-Hydroxy-4-methylbenzaldehyde, Threonine, Sclareol, and Miltirone (details presented in Table 3 and Fig. 4C). According to the results, SMEVs are rich in a range of substances such as terpenoids, sugars, flavonoids, etc., as well as various monomers of *Salvia miltiorrhiza*. Meanwhile, we found that among the 21 active substances of *Salvia miltiorrhiza*, Trijuganone B was at the base peak in the negative ion mode, indicating that SMEVs are rich in Trijuganone B.

**Table 3:** The main monomers of *Salvia miltiorrhiza* in SMEVs

No	Compounds	Formula	Ionization model	RTmed (min)
1	Tanshinone IIA	C19H18O3	[M + H] +	485.7
2	Aethiopinone	C20H24O2	[M + H] +	482
3	Przewaquinone F	C18H16O5	[M + H] +	400.7
4	2-Isopropyl-8-methylphenanthrene-3,4-dione	C18H16O2	[M + H] +	340
5	Miltipolone	C19H24O3	[M + H] +	344.7
6	Danshenxinkun B	C18H16O3	[M + H] +	278.5
7	Deoxyneocryptotanshinone	C19H22O3	[M + H] +	430.3
8	Carnosol	C20H26O4	[M + H] +	433
9	Ferruginol	C20H30O	[M + H] +	457.9
10	Salvianolic acid A	C26H22O10	[M + H] +	326.7
11	Sodium Danshensu	C9H10O5	[M − H] −	363.4
12	Magnesium Lithospermate B	C36H30O16	[M − H] −	348
13	Nortanshinone	C17H12O4	[M − H] −	453.2
14	Hydroxymethylenetanshinquinone	C18H14O4	[M − H] −	399.9
15	Trijuganone B	C18H16O3	[M − H] −	492.1
16	Salvianolic acid C	C26H20O10	[M − H] −	324.7
17	(Rac)-Salvianic acid A	C9H10O5	[M − H] −	363.4
18	2-Hydroxy-4-methylbenzaldehyde	C8H8O2	[M − H] −	313.1
19	Threonine	C4H9NO3	[M − H2O + H] +	48.5
20	Sclareol	C20H36O2	[M + H − 2H2O] +	547.1
21	Miltirone	C19H22O2	[M + Na] +	489.3

### Effects of SMEVs on LPS-induced HUVEC cells

**Fig. 5:**
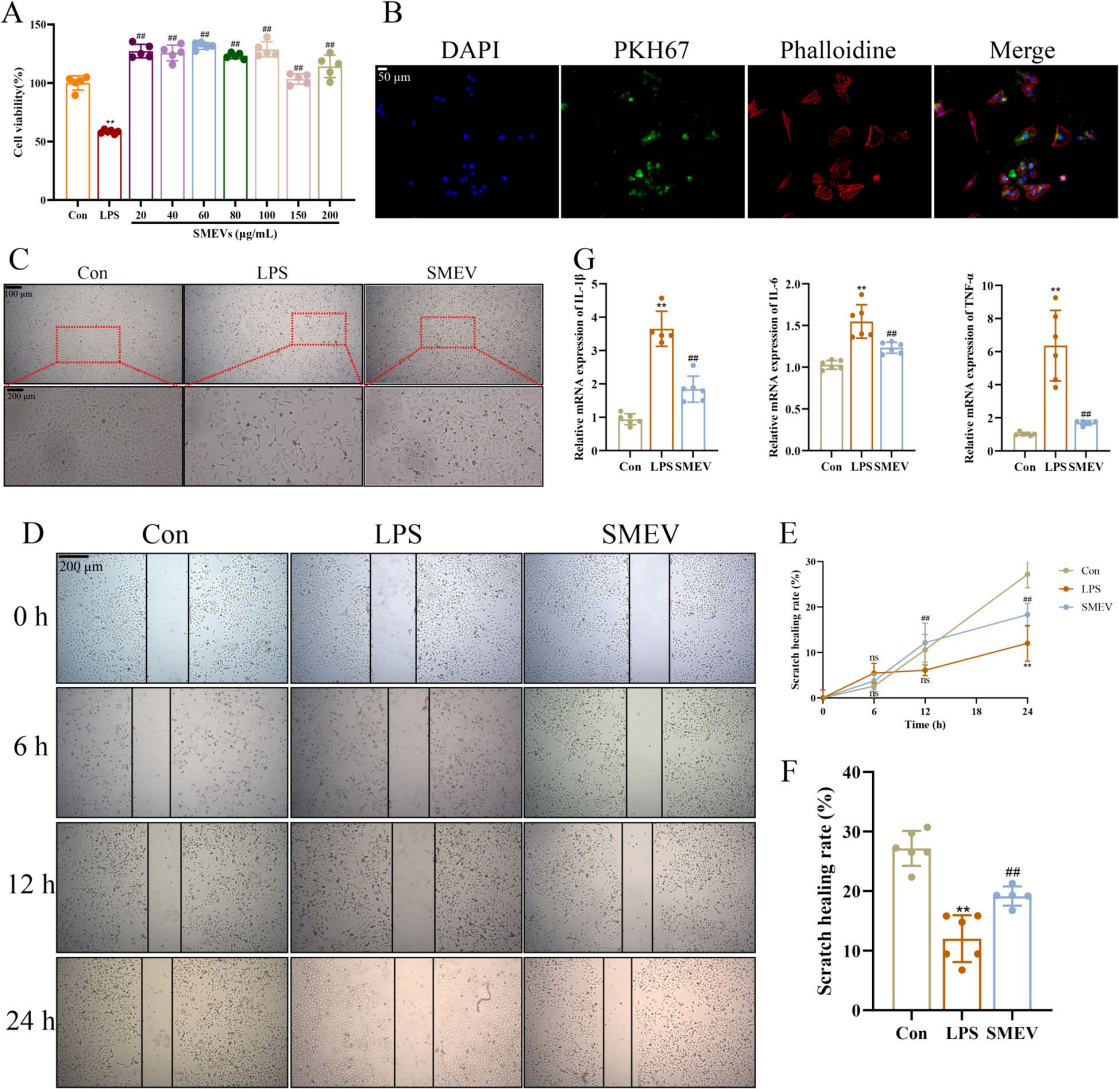
SMEVs Alleviate LPS-Induced HUVEC Inflammation and Vascular Repair. **A** Cell viability evaluation by CCK-8 assay. **B** Uptake of SMEVs by cells. **C** Cell morphology of each group. **D** Wound-healing. **E** Scratch repair rate of cells in different time groups. **F** Scratch repair rate of cells in each group after 24 h

The effects of SMEVs at varying concentrations on the viability of LPS-induced HUVEC cells were assessed by CCK8 assay. It was observed that SMEVs at all concerned concentrations effectively enhanced the cell viability to varying degrees, with the highest cell viability achieved at the concentration of 60 μg/mL. Thus, this concentration was selected for subsequent cell experiments (Fig. 5A). Then, the SMEVs labeled with PKH67 were co-incubated with HUVEC cells, and the results demonstrated that SMEVs were effectively taken up by the cells. Co-localization analysis revealed that they were predominantly localized within the cytoskeletal region (Fig. 5B). Morphological analysis indicated that, compared to the Con group, cells in the LPS group exhibited a reduced cell count within the field of view, along with the presence of cellular debris and vacuolation in a part of cells. Moreover, the cells co-treated with SMEVs showed improvement in cytopathology compared to those treated with LPS alone (Fig. 5C). The scratch assay results yielded no significant differences in wound healing among various groups at 6 h and 12 h, but a notable difference at 24 h (Fig. 5D–F). Subsequently, RT-qPCR was performed to measure the expression of cellular inflammatory factors: in LPS-induced inflammatory cells, SMEVs were found to significantly suppress the mRNA expression of inflammatory cytokines IL-1β, IL-6, and TNF-α (Fig. 5G).

### Protective effects of SMEVs on LPS-induced mice

**Fig. 6:**
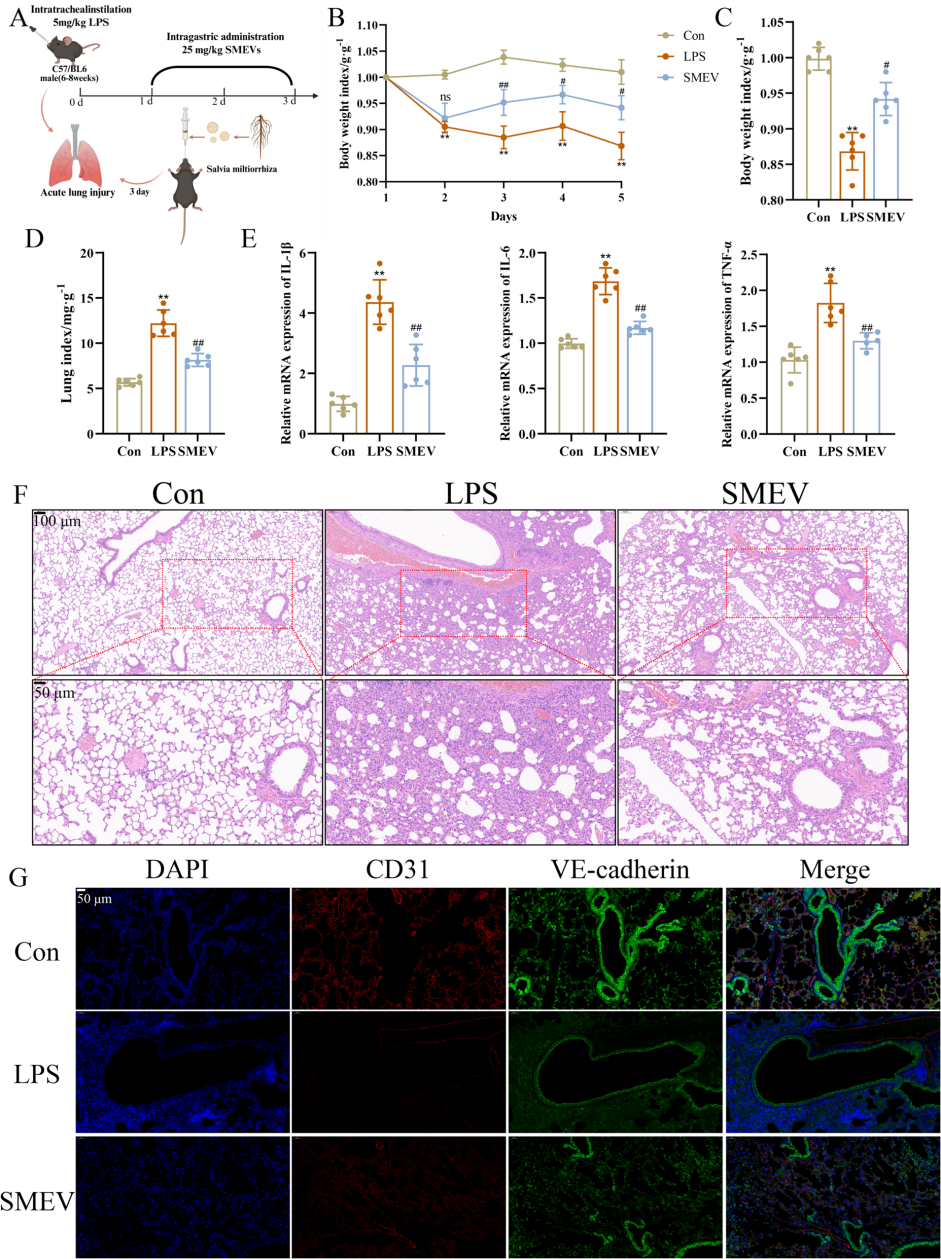
Pulmonary Protective Effects of SMEVs in LPS-induced mouse Model. **A** Schematic illustration of the experimental protocol. **B** Body weight variation profile (n = 6, x̅ ± s). **C** Last Body Mass Index (n = 6, x̅ ± s). **D** Lung index of mice in each group (n = 6, x̅ ± s). **E** mRNA expression levels of IL-1β, IL-6 and TNF-α in lung tissues determined by RT-qPCR (n = 6, x̅ ± s). **F** Representative H&E-stained lung tissue sections. **G** Immunofluorescence analysis of the CD31 (red) and VE-cadherin (green) expression in lung tissues

To evaluate the intervention effects of SMEVs on LPS-induced ALI, in vivo experiments were conducted by following the protocol illustrated in Fig. 6A. The results showed that SMEV treatment significantly restored the weight loss (Fig. 6B, C), reduced the lung index (Fig. 6D), and downregulated the levels of multiple inflammatory factors in LPS-induced lung tissues (Fig. 6E). HE staining revealed that mice in the LPS group exhibited disordered lung tissue structures, featuring thickened alveolar walls, narrowed or collapsed alveolar spaces, extensive exudation, and inflammatory cell infiltration. SMEV intervention markedly improved these pathological changes, mainly manifested by the reduction in alveolar hemorrhage, inflammatory cell infiltration, and interstitial edema (Fig. 6F). Additionally, immunofluorescence results demonstrated that SMEVs increased the expression of the vascular endothelial marker protein CD31 and vascular endothelial barrier marker protein VE-cadherin in the lung tissues of ALI model mice (Fig. 6G).

### Detection of lung microbiota by metagenomic analysis

**Fig. 7:**
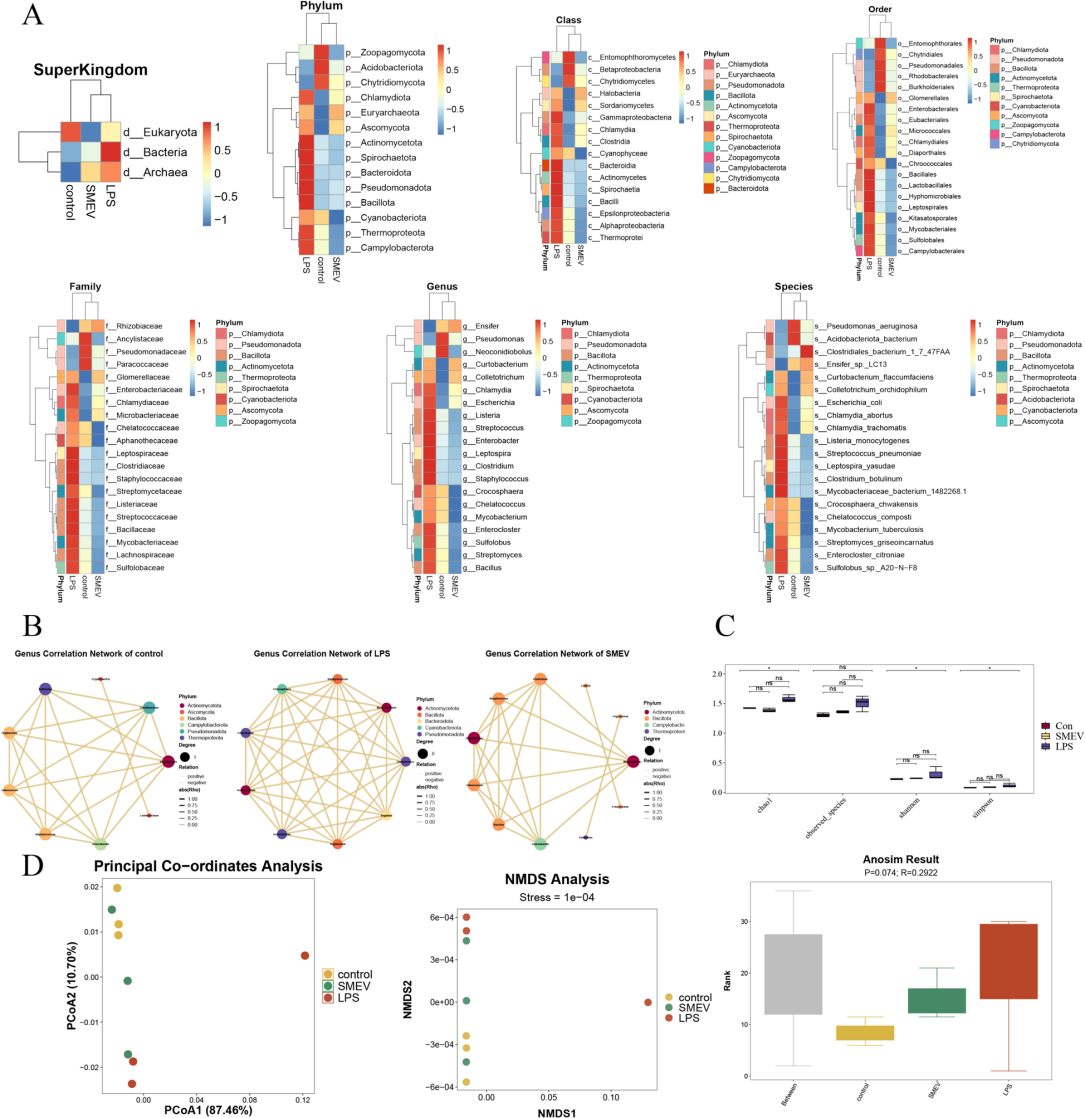
Detection of Microbial Conditions in Mouse Lung Tissues by Metagenomic Sequencing. **A** Statistical analysis of microbial abundance in the lung tissues of mice from different groups. **B** The correlation networks of microbiota within the lung tissues of mice in each group. **C** Alpha diversity analysis. **D** Beta diversity analysis

To elucidate the role of SMEVs in promoting the expression of vascular endothelial barrier-related proteins, we analyzed the lung microbiota composition through metagenomics. It was found that the microbial composition in lung tissues differed to varying degrees across various groups at the levels of Phylum, Class, Order, Family, Genus, and Species (Fig. 7A). Based on the microbial composition in each group of mice, relationships with a default correlation coefficient |rho|> 0.8 were selected to show the species strongly associated with each group through a network diagram (Fig. 7B). Alpha diversity analysis revealed that SMEVs reduced the species richness and diversity of lung microbiota following LPS stimulation, though intergroup differences were not necessarily significant (Fig. 7C). Beta diversity analysis (Fig. 7D) showed that the diversity of lung microbial composition in the Con group was relatively concentrated, while that in the LPS and SMEV groups was relatively dispersed, as evidenced by the Bray–Curtis distance PCoA results. Additionally, NMDS analysis yielded highly accurate and reliable results (stree = 1e-04 < 0.05), which fully reflected the differences in microbial composition between samples. We then performed ANOSIM analysis to assess the statistical significance of the differences in microbial composition between groups, and the results (R = 0.2922, P = 0.074) indicated that the intergroup differences were slightly greater than intragroup differences, but the degree of variation was generally small. Notably, the P value did not reach the commonly recognized significance threshold (*P* < 0.05), though very close, suggesting limited but detectable differences in the microbial composition between groups.

**Fig. 8:**
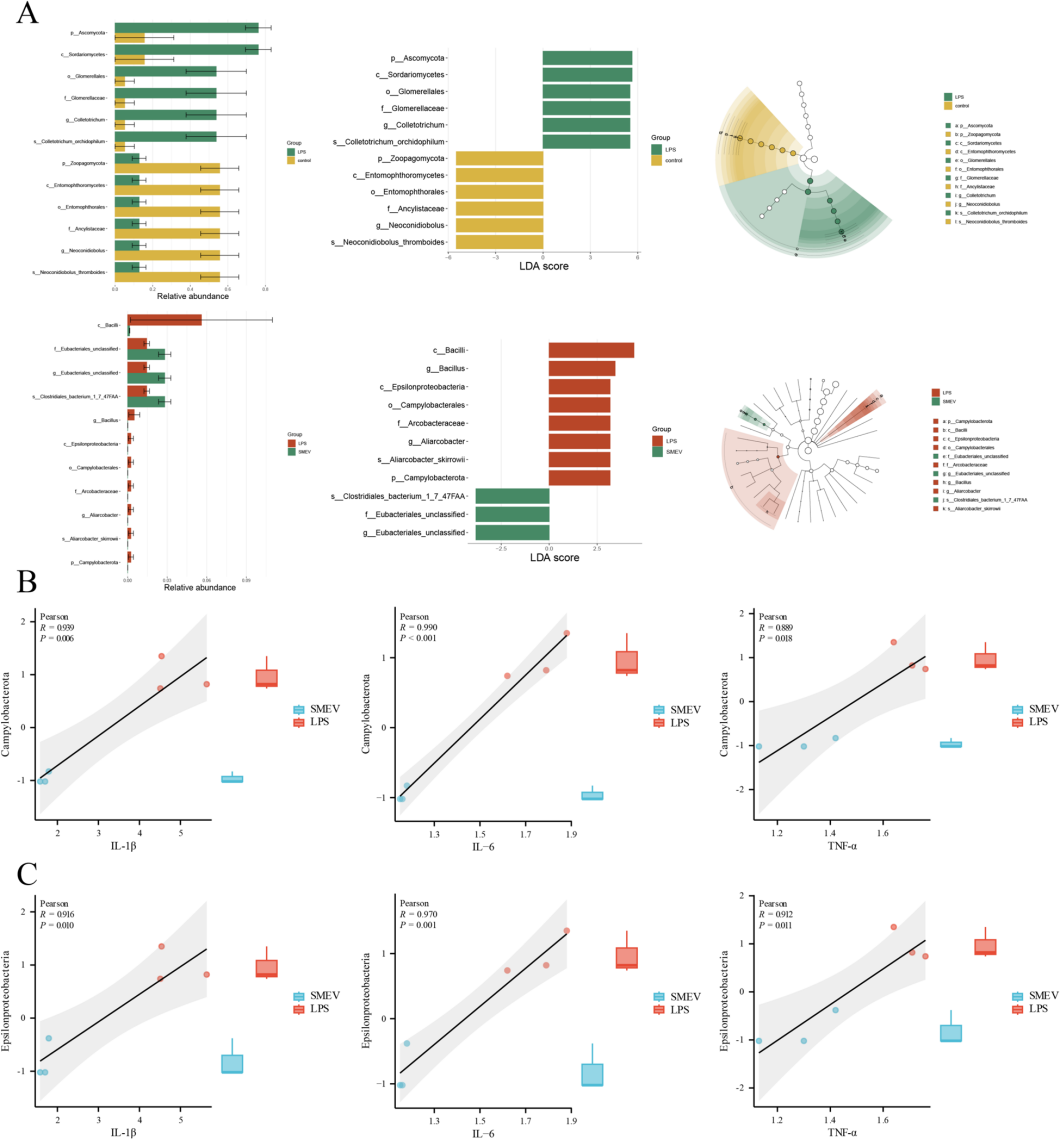
Microbial Differential Screening and Correlation Analysis. **A** LEfSe analysis of lung microbiota in mice from each group. **B** Analysis of the correlation between the Campylobacterota Phylum and inflammatory factors. **C** Analysis of the correlation between the Epsilonproteobacteria Class and inflammatory factors

Further, LEfSe analysis was performed to identify key differential microorganisms between groups (Fig. 8A), and significant differences were revealed in the lung microbial composition between the Con and LPS groups, as well as between the LPS and SMEV groups. Additionally, LEfSe analysis based on LDA scores further confirmed the presence of a range of characteristic genera in the lung tissues of the Con group versus the LPS group, and in the LPS group versus the SMEV group. Specifically, the main differences between the Con and LPS groups were concentrated in the Ascomycota and Zoopagomycota Phyla, while the main differences between the LPS and SMEV groups were concentrated in the Campylobacterota Phylum, and the Epsilonproteobacteria Class. Subsequently, through Pearson analysis, we conducted a correlation analysis between the differential microbial communities (i.e., Campylobacterota Phylum and Epsilonproteobacteria Class) and the mRNA expression of inflammatory factors in the lung tissues of the LPS and SMEV groups (Fig. 8B, C). The results highlighted a strong correlation between both Campylobacterota and Epsilonproteobacteria and the mRNA expression of inflammatory factors.

## Discussion

ALI is a severe clinical condition—disruption of the microvascular barrier represents a critical pathophysiological process in this disease. Currently, corticosteroids or lung protective ventilation strategies are predominantly used in the clinical treatment of ALI, but there are still many limitations, making development of new therapeutic agents critically important [30].

It has been reported that ALI-induced vascular endothelial injury can damage the pulmonary microvascular barrier, thereby increasing pulmonary microvascular permeability, inducing capillary leakage and edema, exacerbating inflammatory injury, and ultimately resulting in high morbidity and mortality [31, 32]. This complex process constitutes the primary pathogenesis of ALI. The microvascular endothelial barrier, which is composed of a continuous monolayer of endothelial cells between the blood and interstitial tissue, plays a key role in regulating multiple physiological functions, including the transport of water and nutrients, vascular tone, aggregation and adhesion of inflammatory mediators, as well as hemostasis and thrombosis [33, 34]. Adherens junctions are a major component of endothelial junctions, consisting of transmembrane proteins that interact with adjacent cells to maintain the integrity of the microvascular endothelial monolayer and to regulate its barrier function [35]. VE-cadherin, the primary adherens junction protein in vascular endothelial cells that connects intracellularly to the cortical F-actin cytoskeleton, is a dominant factor in maintaining the stability of adherens junctions and the integrity of endothelial barrier [36]. Once inflammatory injury occurs, VE-cadherin tends to undergo a series of reactions including phosphorylation, destabilization, and internalization, which can directly impair the microvascular endothelial barrier and exacerbate inflammation, creating a vicious cycle [37]. Meanwhile, ALI-induced inflammation can lead to disruption of the microvascular endothelial barrier, further amplifying the host’s inflammatory condition [38]. The systemic inflammatory response induced by ALI is then rapidly activated, triggering a fast and effective defense mechanism to mitigate the microvascular endothelial barrier permeability, while promoting the production and migration of monocytes, macrophages, and other immune cells to resist pathogenic microorganisms and endotoxin invasion [39, 40]. Concurrently, ALI can also cause an imbalance in microvascular endothelial barrier permeability by triggering the release of excessive inflammatory mediators, which in turn aggravates microvascular leakage, ultimately resulting in circulatory failure accompanied by multi-organ dysfunction [41, 42]. Overall, interaction among the microvascular endothelial barrier, adherens junctions, and excessive inflammatory mediators plays a pivotal role in the pathophysiology of ALI, suggesting that protecting microvascular endothelial adherens junctions may serve a potential target and strategy for the prevention and treatment of ALI.

In this study, after determining that SMEVs are safe, non-toxic, and can be taken up by HUVEC cells, we further confirmed through a series of experiments that SMEVs can not only alleviate inflammatory responses in LPS-induced HUVEC cells but also mitigate pulmonary edema, effusion, and inflammatory responses in ALI model mice. For HUVEC cells, SMEVs may even promote vascular wound repair upon LPS stimulation. Animal experiments reached similar findings: immunofluorescence results showed that SMEV treatment promoted the expression of VE-cadherin in the lung tissues of LPS-induced ALI mice. These evidences provide strong support on the protective role of SMEVs in maintaining the adherens junctions of pulmonary microvascular endothelial barrier to alleviate inflammation. In addition to the direct protective effects of SMEVs on endothelial barrier integrity, the miRNA cargos carried by SMEVs may also play a crucial role in regulating inflammatory responses and vascular repair. Earlier studies indicate that EVs-derived miRNAs are capable of alleviating lung injury by enhancing the vascular endothelial barrier stability or suppressing excessive inflammatory responses through modulation of inflammatory signaling pathways [43, 44]. This suggests that the miRNAs in SMEVs can synergistically mitigate inflammatory responses and microvascular leakage in ALI through mechanisms involving multiple targets and pathways. Further elucidation of these specific mechanisms may be achieved by miRNA sequencing and target gene validation in future research.

SMEVs demonstrate unique advantages in ALI treatment compared to conventional *Salvia miltiorrhiza* extracts. While exhibiting anti-inflammatory, antioxidant, and vasculoprotective effects, the clinical application of conventional extracts (e.g., tanshinone IIA) is usually limited by poor bioavailability and nonspecific drug distribution [45, 46]. In contrast, SMEVs, as natural nanocarriers, may achieve better therapeutic outcomes with increased drug delivery efficiency, reduced systemic toxicity, and more precise targeting of pulmonary microvascular endothelial cells. Furthermore, although plant-derived EVs (e.g., those from *Artemisia annua*, *Platycodon grandiflorum*, and *Ginseng*) have been extensively investigated for their anti-inflammatory and immunomodulatory properties [21, 29, 47], SMEVs may possess superior advantages in maintaining vascular barrier integrity and modulating inflammatory responses owing to their unique bioactive components (e.g., Tanshinone IIA, Salvianolic acid A, Salvianolic acid C, Sodium Danshensu, Danshenxinkun B), which deserves in-depth research.

Through the ages, the lungs were believed to be sterile until technological advancements revealed the existence of a lung microbial community, in which the microorganisms suspended or attached to airborne particulate matters are considered the primary source of microbiota. After these microorganisms disperse into the lungs, the microbiota achieves a delicate balance through reproduction, migration, and elimination [48]. Under normal conditions, the microbial reproduction rate in the lungs maintains at a generally low level, while the migration and elimination rates are much higher, resulting in a highly diverse microbiota in healthy states, influenced by the surrounding environment. Nonetheless, the composition and dominant species of the lung microbial community change dynamically with the state of the lungs [49, 50]. On the other hand, the mechanisms of ALI are complex and varied, involving the release of inflammatory cytokines and disruption of the pulmonary microvascular barrier. It has been shown that mechanisms resembling cell infection, invasion, and lysis caused by respiratory microbial secreted factors may compromise the integrity of the vascular endothelial cell layer. In influenza virus-induced ALI, viruses are found to disrupt the microbial balance by damaging the lung barrier system and inhibiting alveolar cells from maintaining surfactants [51]. Additionally, experiments focusing on IL-6-induced ALI showed that the ALI-induced changes in the lung microbiota actually increased the host’s susceptibility to lung injury under repeated damage conditions [49]. Meanwhile, in LPS-induced ALI mice, the lung microbiota was revealed to play complex roles in vascular barrier integrity, inflammation, immune continuum activity, and antimicrobial host defense [52, 53], suggesting that the regulatory status of lung microbiota may serve as a potential biomarker for the severity of lung injury. In this study, we employed the metagenomic sequencing technology to systematically evaluate the effects of SMEVs on the microbial community in mouse lung tissues. It was found that the LPS group exhibited increased microbial count and richness at all taxonomic levels including Phylum, Class, Order, Family, Genus, and Species, as compared to the Con group, whereas SMEV treatment reversed this trend. Taking the Genus level as an example, the bacteria that were most altered by LPS stimulation were g-Listeria, g-Streptomyces, and g-Streptococcus. Specifically, g-Listeria is a pathogenic bacterium that can cause meningitis and sepsis in severe cases; g-Streptococcus is another significant pathogenic bacterium that can cause various diseases such as scarlet fever, erysipelas, pneumonia, and meningitis; and g-Streptomyces, as an important microbial resource, can be used clinically as antibiotics by leveraging its production of various secondary metabolites, but can also cause skin abscesses, fever, arthritis, pneumonia, bacteremia, and sepsis in immunocompromised individuals. Alpha diversity analysis indicated that SMEV treatment showed a potential to reduce the diversity and richness of lung microbiota after LPS stimulation, though the intergroup differences did not reach a statistical significance level, suggesting that SMEVs may alleviate LPS-induced inflammatory responses by regulating the composition of lung microbial community. Beta diversity analysis demonstrated that the microbial composition in lung tissues of the Con group was relatively concentrated, while both the LPS and SMEV groups exhibited a higher level of dispersion. The reliability of these results was further supported by the high accuracy of NMDS analysis (stress = 1e-04 < 0.05). ANOSIM analysis revealed that the intergroup differences were slightly greater than the intragroup differences (R = 0.2922), but did not reach the commonly recognized significance level (P = 0.074), suggesting limited but detectable differences in microbial composition between different groups. Subsequently, through LEfSe analysis, we further identified the key differential microorganisms between groups. It was found that the differences between the Con and LPS groups were mainly concentrated in the Ascomycota and Zoopagomycota Phyla, while the differences between the LPS and SMEV groups were concentrated in the Campylobacterota Phylum, and the Epsilonproteobacteria Class. Moreover, Pearson analysis revealed a strong correlation between these two differential microbial communities and the mRNA expression of inflammatory factors in lung tissues. We speculate that these differences in microbial composition may play an important role in the mechanisms of LPS-induced inflammatory responses and the protective effects of SMEVs exerted via the vascular barrier system. As evidence supporting our hypothesis, we observed that SMEVs not only reduced the LPS-induced enrichment in pathogenic bacteria (e.g., o-Campylobacterota, c-Epsilonproteobacteria) in lung tissues but also upregulated the VE-cadherin expression, suggesting a close association between microbial variation and vascular barrier restoration. Earlier studies have demonstrated that certain pathogens can exacerbate endothelial injury by releasing toxins or activating the TLR/NF-*κ*B pathway [54, 55]. In contrast, SMEVs may directly neutralize bacterial components by leveraging their miRNAs or the unique bioactive compounds from *Salvia miltiorrhiza* (e.g., Tanshinone IIA, Salvianolic acid A, Salvianolic acid C, Sodium Danshensu, Danshenxinkun B), thereby reshaping the lung microbiota, attenuating pro-inflammatory bacterial populations, and directly or indirectly promoting the VE-cadherin stabilization and barrier repair. Whether SMEVs exert their protective effects in ALI by regulating a specific microbiota or its metabolites (e.g., SCFAs, tryptophan derivatives), thereby influencing the phosphorylation status of endothelial junctional proteins, needs to be confirmed by further investigations.

In summary, this study revealed that SMEVs exert a therapeutic effect on ALI by regulating the composition and diversity of the lung microbial community and improving the function of the lung tissue vascular barrier system. Future research is expected to further explore the functions of these differential microorganisms and their interaction with the host immune system, aiming to provide new targets and strategies for the treatment of pulmonary inflammatory diseases.

## Conclusion

**Fig. 9:**
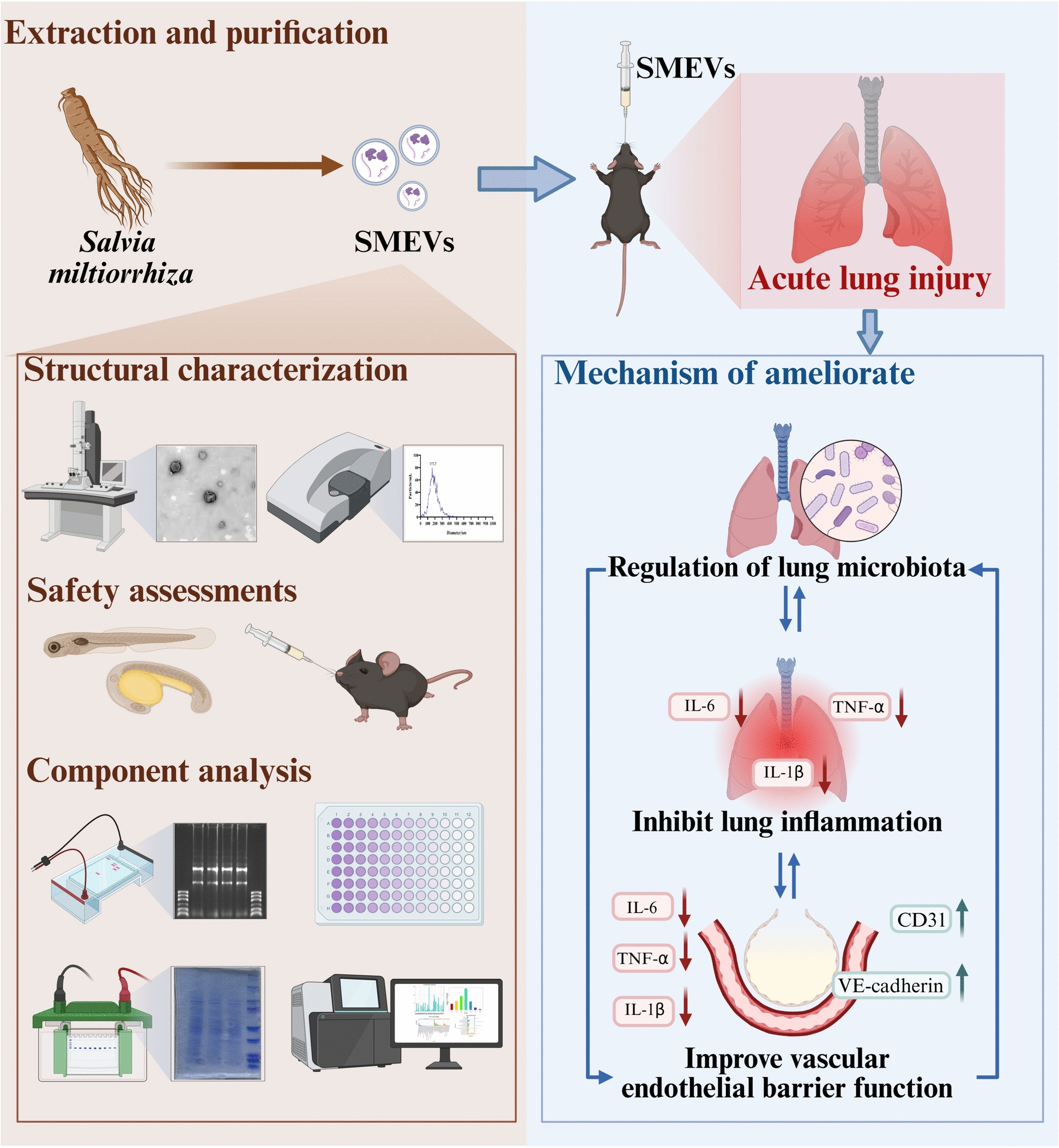
Schematic Illustration of SMEV-Mediated Alleviation of ALI

This study, through systematic experimentation and metagenomic analysis, elucidated the significant role of SMEVs in mitigating ALI, as well as the underlying mechanisms. Our findings demonstrate that SMEVs may ameliorate and repair the pulmonary vascular barrier system, thereby alleviating inflammatory damage, potentially by regulating key components and functions of the lung microbiota. This deepens our understanding of the interaction between the pulmonary vascular barrier system and the host microbiota. Future research is expected to further clarify the interaction among SMEVs, the lung microbiota, and the host immune system, hoping to yield better-targeted approaches for the prevention and management of ALI (Fig. 9).

## Data Availability

All data generated or analyzed during this study are included in this published article.

## Abbreviations

ALI: Acute lung injury
EV: Extracellular vesicle
HE: Hematoxylin–eosin
HUVEC: Human umbilical vein endothelial cells
IL-1*β*: Interleukin-1*β*
IL-6: Interleukin-6
LPS: Lipopolysaccharide
NTA: Nanoparticle tracking analysis
SMEV: *Salvia miltiorrhiza*‐Derived Extracellular Vesicle
TEM: Transmission Electron Microscope
TNF-α: Tumor necrosis factor-α
VE-cadherin: Vascular endothelial-cadherin
